# The Influence of Mission Valence on Faculty’s Voice Behavior: The Role of Thriving at Work and Servant Leadership

**DOI:** 10.3390/bs14121214

**Published:** 2024-12-18

**Authors:** Xi Liu, Zhixia Chen, Mei Sun

**Affiliations:** 1 College of Public Administration, Huazhong University of Science and Technology, Wuhan 430074, China; lx5467@hust.edu.cn (X.L.); zhxchen@hust.edu.cn (Z.C.); 2 School of Humanity and Law, Henan Agricultural University, Zhengzhou 450046, China

**Keywords:** mission valence, faculty’s voice behavior, thriving at work, servant leadership

## Abstract

Faculty’s voice behavior is crucial in promoting institutional reform and sustainable development in higher education institutions. However, there is still significant room for exploration regarding how to effectively stimulate such behavior among faculty. This study, based on data collected from 630 Chinese university faculty, investigates the conditions under which mission valence can promote voice behavior in higher education settings. The study involved constructing a moderated mediation model, with thriving at work as the mediator and servant leadership as the moderator, to explore the relationship between mission valence and faculty’s voice behavior. The results reveal that mission valence has a significant positive impact on faculty’s voice behavior in higher education and indirectly promotes such behavior through the mediating role of thriving at work. Furthermore, it was found that servant leadership plays a significant moderating role in the relationship between thriving at work and faculty’s voice behavior, enhancing the overall mediation mechanism. This study extends our understanding of the relationship between mission valence and faculty’s voice behavior in the context of Chinese higher education and provides practical insights into strategies for promoting faculty’s voice behavior.

## 1. Introduction

In the context of deepening reforms in higher education, faculty play a critical role in advancing scientific research, generating innovative outcomes, and cultivating high-quality talent, thus driving the transformation and development of higher education institutions [1,2]. In recent years, while there has been a growing call for greater faculty participation in university governance, challenges persist, such as a lack of responsibility and mission among some faculty, low participation willingness, and poor participation effectiveness [3]. Faculty’s voice behavior is an important avenue for their involvement in university management and organizational change. By integrating the wisdom and strength of faculty across disciplines, it is possible to build a high-quality, professional, and innovative teaching force, optimize quality management in higher education institutions, and foster technological innovation and talent cultivation, which are vital for the democratization of university governance [4]. Hence, effectively stimulating faculty’s voice behavior is a pressing issue in the current human resource management practices of higher education institutions. Previous studies have found that individual and organizational-level variables, such as personality traits, leadership styles, and organizational context, can significantly influence faculty’s voice behavior [4,5,6,7,8,9]. Furthermore, while research has identified the importance of individual psychological and cognitive variables in stimulating voice behavior [10], there remains room for further exploration into the impact of individual cognitive factors on faculty’s voice behavior.

Mission valence represents the extent to which faculty are attracted to the organizational mission and perceive its importance [11]. According to conservation of resources theory, mission valence is a form of positive energy resource [12]. Voice behavior requires a high level of resource investment from individuals [13,14], and mission valence can enhance individuals’ psychological and emotional resources, facilitating extra-role and proactive behaviors [15,16]. With the implementation of a series of education activities upholding the spirit of “remain true to our original aspiration and keep our mission firmly in mind” in Chinese universities, mission-oriented education has had a profound impact on faculty organizational identity and sense of belonging, prompting them to develop a renewed understanding and perception of the mission of higher education institutions. This reinforced mission valence may become a key driver of faculty’s voice behavior in higher education institutions. Therefore, identifying “whether high mission valence among university faculty will lead to increased voice behavior” is crucial in addressing the issue of faculty participation in university governance.

The voluntary and challenging nature of voice behavior, along with the associated risks, not only requires individual resources but also emphasizes their work state [17]. In the educational context, thriving at work is a positive cognitive and emotional state experienced by faculty [18]. According to the broaden-and-build theory of positive emotions, a positive psychological state can broaden individuals’ cognition and help them acquire and enhance various resources, including physical and psychological ones [19,20]. Therefore, faculty thriving at work provides sufficient resource support for the occurrence of voice behavior and is an important driver of such behavior. At the same time, thriving at work is influenced by individual work resources [21], and public sector employees’ perception and recognition of the organizational mission can provide positive emotional resources for their work state [22], suggesting a potential positive correlation between mission valence and faculty thriving at work. Additionally, previous studies have found that individuals with high levels of thriving at work are more willing to invest time and energy in activities related to change [23]. Furthermore, research has shown that thriving at work mediates the relationship between transformational leadership and subordinate innovative behavior [24], between perceived respect and innovative behavior [25], between time pressure and innovative behavior [26], and between employee career growth opportunities and career commitment [27]. As previously mentioned, in the educational context, voice behavior is a proactive behavior that faculty engage in to benefit departmental development, while mission valence is their cognitive experience based on their perception and understanding of the organizational mission, and thriving at work reflects their ongoing positive work state. Therefore, this study attempts to explore the mediating role of thriving at work in the relationship between mission valence and faculty’s voice behavior in higher education institutions.

Moreover, the environment in which faculty find themselves can influence their psychological and cognitive processes as well as their behavior [28,29]. Previous research has explored the impact of contextual factors on voice behavior [30,31,32]. Servant leadership is a leadership style that emphasizes humanism, focusing on leaders’ service-oriented spirit centered on employees [33]. According to the conservation of resources theory, servant leadership, as a form of collective leadership, is an important organizational resource for faculty, potentially influencing their voice behavior [34]. Faculty’s voice behavior is often accompanied by the risk of leadership rejection and challenges, and whether faculty engage in voice behavior is largely influenced by departmental leadership. If leaders encourage faculty to have a voice, providing them with space to voice their suggestions, faculty are more likely and more willing to engage in voice behavior [9]. In other words, the relationship between thriving at work and faculty’s voice behavior in higher education institutions may vary depending on the leadership style, with servant leadership potentially playing a buffering or reinforcing role in the influence of thriving at work on faculty’s voice behavior. Therefore, this study attempts to introduce servant leadership as a moderating variable to explore the boundary conditions under which mission valence influences faculty’s voice behavior in higher education institutions.

In summary, based on the conservation of resources theory, the broaden-and-build theory of positive emotions, and existing research, this study examines the internal mechanisms by which mission valence influences faculty’s voice behavior in higher education institutions, using university faculty as the research sample. This study aims to contribute in the following ways: first, by validating the positive impact of mission valence on faculty’s voice behavior in higher education and analyzing the mediating role of thriving at work in this relationship. This study empirically validates the motivational function of mission valence in stimulating faculty’s voice behavior. Second, by examining the moderating effect of servant leadership on the relationship between thriving at work and faculty’s voice behavior in higher education, this study explains the interaction between individual and organizational factors influencing faculty’s voice behavior. In summary, this study constructs a moderated mediation model to deeply analyze the process mechanisms and boundary conditions of the impact of mission valence on faculty’s voice behavior in higher education, with the aim of unveiling the “black box” of the relationship between mission valence and faculty’s voice behavior, enriching the understanding of mission valence’s effectiveness, and providing theoretical and practical insights for enhancing mission valence among university faculty and stimulating their voice behavior.

## 2. Literature Review and Hypotheses

### 2.1. Mission Valence and University Faculty’s Voice Behavior

The concept of mission valence was first proposed by Rainey and Steinbauer, who, drawing on the concept of valence in expectancy theory, defined it as the psychological perception and value judgment of employees regarding the importance and attractiveness of their organization’s mission. It reflects the degree to which the organizational mission motivates individuals [35]. Previous research has explored the outcome variables of mission valence, finding that it can influence task significance [12], organizational commitment [16], stakeholder support willingness [36], turnover intention [15], and other employee attitudes, and can effectively stimulate work motivation through the importance of work goals [11]. Additionally, mission valence can significantly enhance proactive and extra-role behaviors among employees [15].

The concept of voice behavior originated from Hirschman’s EVL model, where it referred to employees’ appeals to management for change due to dissatisfaction with their work [37]. Later, LePine and Van Dyne defined voice behavior as employees proactively proposing ideas or suggestions related to work in order to improve their department or organization [38]. In the educational context, as an important form of faculty’s proactive participation in organizational management, voice behavior is a constructive and challenging extra-role behavior [39], playing a vital role in the transformation and development of university departments [8,40]. Previous research has explored the antecedents of individual voice behavior, finding that employees’ attitudes and perceptions of the organization and work are important factors in stimulating voice behavior [40]. For example, employees’ constructive change responsibility positively influences their voice behavior [41], and workgroup identification positively influences their voice behavior [42]. Research on faculty’s voice behavior has also found that university faculty’s organizational identification positively influences their voice behavior [7]. Based on this, this study hypothesizes that mission valence, as a measure of faculty’s perception and evaluation of the importance of the organizational mission, will lead to more expression of their views and opinions regarding their work, i.e., more voice behavior.

Conservation of resources theory describes the process of interaction between resources and individuals in their social environment [43]. The theory emphasizes that individuals, in order to avoid resource depletion, will make every effort to acquire, preserve, and protect resources they consider valuable [44]. This theory provides an analytical framework for understanding the relationship between mission valence and university faculty’s voice behavior.

Firstly, according to conservation of resources theory, when individuals possess abundant resources, they are more willing and able to invest resources to expand their resource pool, thereby achieving a resource gain spiral [45]. Mission valence is a form of positive energy resource [12]. In the educational context, high mission valence among university faculty indicates a high degree of alignment between their personal values and the department’s mission and organizational goals [46]. High mission valence not only enhances faculty’ organizational commitment [16] but also helps increase their recognition and support of the educational mission of their department, thereby increasing their trust and reliance on the department as an emotional resource, resulting in a relatively abundant resource pool. On this basis, to acquire more resources and achieve resource gains to cope with future risks, faculty will continue to invest resources. Voice behavior is an important means by which individuals invest resources to gain valuable resources [47,48], as it helps faculty gain recognition from the organization and superiors, receive opportunities for promotion, and access other resources. Therefore, university faculty with high mission valence, driven by their emotional attachment to the department and their motivation to preserve and expand their personal resources, will increase their perception of the importance of work goals [11] and engage in more voice behavior to promote educational reform and help achieve the university’s mission, thereby acquiring more resources. Additionally, as previously mentioned, high mission valence among university faculty indicates a high degree of alignment with the university’s educational mission and goals, and a stronger sense of mission. A strong sense of mission is also an important personal resource [49], and faculty with a strong sense of mission often exhibit high levels of professional self-efficacy [50], which can stimulate their voice behavior.

Secondly, according to conservation of resources theory, the growth of an individual’s resource pool can help alleviate work pressure and stimulate positive work motivation [44]. With the increasing demand for building world-class universities, the resulting research and teaching assessments and task–role balance create additional work burdens and pressure for university faculty [51]. Excessive teaching hours and research assessment requirements make it difficult for university faculty to derive a sense of personal accomplishment and work significance from their work [52], leading to burnout and even turnover intentions [53]. Mission valence is the evaluation of the importance and social significance of the organizational mission and goals by university faculty. Faculty with high mission valence are more likely to believe that the educational mission and talent cultivation goals of universities have significant social significance, and they will thus be convinced that their work positions and tasks are valuable in achieving the department’s mission and goals [12], prompting them to engage in extra-role behaviors like voice behavior [15]. In other words, mission valence can provide positive psychological and emotional resources for university faculty, reducing emotional exhaustion and thus lowering work burnout [22], enhancing work happiness and enthusiasm, boosting work motivation [11], and encouraging them to engage in voice behavior to demonstrate the significance and value of their work. Based on the above, this study proposes Hypothesis 1:

**Hypothesis** **1.** *Mission valence has a significant positive impact on university faculty’s voice behavior.*

### 2.2. Mission Valence and Thriving at Work

Thriving at work refers to a psychological state and subjective work experience in which employees simultaneously experience “vitality” and “learning” [18], with vitality representing a state of enthusiasm and liveliness at work [54], and learning representing a state of acquiring new knowledge or skills [55]. These two states help employees quickly adapt to changing work environments and promote both work and personal development [18]. Currently, research on the factors influencing thriving at work is still relatively scarce, with existing studies suggesting that external resource elements such as work resources provided by the organization and management context are important factors influencing employee thriving at work [18]. However, research on the internal resource elements that nourish thriving at work is still relatively limited. Previous studies have suggested that individual internal resources, such as positive emotional traits, self-control, and self-esteem, can significantly enhance faculty’s thriving at work [56], and mission valence is also an important internal energy resource for faculty [12], reflecting their perception and evaluation of the educational mission. Therefore, mission valence among university faculty may be one of the factors promoting their thriving at work.

According to the resource gain spiral mechanism in conservation of resources theory, abundant initial resources facilitate the subsequent acquisition and growth of resources, enabling individuals to accumulate resources continuously [45]. Mission valence among university faculty reflects their perception of the social significance and importance of the university’s educational mission [46], contributing to the formation of high vitality and high learning states. Specifically, from the vitality dimension, high mission valence indicates that the educational mission and talent cultivation values of the university have been effectively communicated to and internalized by faculty, reinforcing their perception of work significance and sense of responsibility, injecting additional energy resources [12], increasing their work enthusiasm and vitality, and filling them with passion in their work. From the learning dimension, in the face of increasingly demanding work requirements, higher mission valence among university faculty leads to stronger recognition of the university’s mission. Therefore, to better fulfill administrative, teaching, and research responsibilities, university faculty engage in continuous self-learning to acquire the knowledge, skills, and information resources needed for their work, meet work demands, and enhance their professional competitiveness, providing ample resources for achieving and maintaining a thriving state at work [21]. Based on the above analysis, this study proposes Hypothesis 2:

**Hypothesis** **2.** *Mission valence has a significant positive impact on university faculty’s thriving at work.*

### 2.3. The Mediating Role of Thriving at Work

This study hypothesizes that thriving at work has a positive impact on university faculty’s voice behavior. As mentioned earlier, thriving at work is a psychological state in which employees simultaneously experience “vitality” and “learning” [18], and this positive state can enhance university faculty’s willingness and ability to engage in voice behavior. First, vitality represents a positive emotional state. According to the broaden-and-build theory of positive emotions, positive emotions not only strengthen individuals’ resources through building mechanisms but also improve cognitive flexibility and creativity [57]. The resource gains and cognitive enhancement brought about by vitality are key factors for the occurrence of voice behavior. Furthermore, voice behavior is an extra-role behavior that requires spontaneous and persistent engagement; when faculty are filled with enthusiasm and vitality in their work, they are more likely to express their ideas and suggestions. Therefore, the vitality experienced by university faculty at work provides support for their willingness to use their voice, which is essential for the continuation of voice behavior. Second, voice behavior requires a continuous supply of new knowledge and skills, and the continuous learning and growth experienced by faculty in their daily work help them identify problems and propose new ideas [58], equipping them with the foundation for voice behavior. Lastly, empirical research findings also support the potential relationship between thriving at work and voice behavior among university faculty. For example, Fan et al. (2022) [59] found that thriving at work, through psychological safety, can lead to voice behavior. Sheng and Zhou’s (2022) [60] pointed out that the vitality and learning elements associated with thriving at work provide employees with more energy and a broader perspective, thereby stimulating voice behavior. Liu and Zhou (2024) [61] further discovered that employees’ thriving at work not only promotes voice behavior but also helps them gain work resources, further facilitating continued thriving at work. Therefore, this study proposes Hypothesis 3:

**Hypothesis** **3.** *Thriving at work has a significant positive impact on university faculty’s voice behavior.*

According to the social embeddedness model of thriving at work and existing research, thriving at work can serve as a bridge linking work resources and individual behaviors [18]. Given this, this study posits that thriving at work can effectively transmit the motivational function of mission valence on university faculty’s voice behavior. Specifically, first, as a form of positive energy resource, mission valence can enhance university faculty’s identification with the university’s organizational mission, thereby promoting and sustaining thriving at work among faculty, facilitating the acquisition of work resources, and boosting work vitality and learning. Second, thriving at work, as a positive psychological state, with its high vitality and high learning dimensions, can strengthen faculty’s willingness and ability to engage in voice behavior, motivating them to propose more suggestions for the future development of departments and students, improving educational quality and effectiveness. Based on this, this study proposes Hypothesis 4:

**Hypothesis** **4.** *Thriving at work mediates the relationship between mission valence and university faculty’ voice behavior.*

### 2.4. The Moderating Role of Servant Leadership

The occurrence of faculty’s voice behavior relies on the joint influence of various factors, and the interaction between individual factors and contextual factors offers new insights into better explaining individual voice behavior in complex contexts [62]. Therefore, after establishing the mediating effect of faculty’s individual cognition and work state on voice behavior, it is necessary to further consider the interaction between contextual factors and individual factors to more comprehensively explain the internal mechanisms of faculty’s voice behavior in higher education. According to conservation of resources theory, the resources that individuals seek to obtain are often influenced by specific ecological contexts, known as resource caravan passageways, which can nurture, foster, and create resources or restrict, weaken, and undermine them [63]. Theoretically, servant leadership emphasizes prioritizing others over self-interest and is a people-oriented leadership style [64]. This style can nurture and foster the maintenance and utilization of individual energy and work resources, thus serving as an important resource caravan passageway in organizations. Additionally, research has found that university faculty are more likely to engage in voice behavior in contexts with high levels of servant leadership [65]. Therefore, this study posits that servant leadership, as a resource caravan passageway, can provide crucial contextual support for the transformation of thriving at work into voice behavior, with university faculty’s performance in the voice process largely depending on whether the organizational climate created by servant leadership is caring, encouraging, and inclusive.

Specifically, servant leadership can create a “service-oriented climate” in organizations, characterized by support, assistance, and encouragement [66,67]. University faculty with high thriving at work are more likely to perceive and be influenced by this positive voice climate, thereby encouraging more voice decisions and facilitating the activation of their voice behavior. On one hand, servant leadership in higher education institutions promotes faculty’s participation in management through empowerment, boosting their confidence and maintaining their work vitality. At the same time, the trust and support provided by servant leadership encourage faculty to deepen their understanding of work content through continuous learning, thereby enhancing their thriving at work. In other words, high levels of servant leadership enable university faculty to receive more supportive resources such as respect, emotional support, information, and skills. According to conservation of resources theory, resources have a motivating effect on positive behavior [68]; thus, an increase in psychological and material resources among university faculty contributes to their confidence and ability to engage in voice behavior, thereby promoting their voice behavior. On the other hand, servant leadership fosters good communication with faculty, understanding of their personal needs and development goals through care and listening, and providing the necessary services, thereby encouraging their participation in management and unlocking their potential [69]. This supportive organizational climate can alleviate the negative emotions, such as anxiety, that university faculty may experience due to the risks associated with voice behavior [70], enhance their psychological safety, reduce the pressure from potential resource loss, and the leadership support and inclusiveness can stimulate university faculty’s sense of responsibility to reciprocate, prompting them to proactively express their views on departmental decisions and offer suggestions for decision optimization, i.e., engage in more voice behavior. Based on this, it is hypothesized that when servant leadership is high, the increase in resource pool and reduction in resource loss pressure under high thriving at work enable university faculty to not only be willing to but also dare to engage in voice behavior. Conversely, when servant leadership is low, driven by a logic of responsibility avoidance, university faculty tend to remain silent to preserve existing resources and avoid resource loss. Even under high thriving at work, they may want to give voice but lack the courage to do so, resulting in less voice behavior. Based on the above, this study proposes Hypothesis 5:

**Hypothesis** **5.** *Servant leadership positively moderates the relationship between thriving at work and university faculty’s voice behavior, i.e., the higher the level of servant leadership, the stronger the impact of thriving at work on university faculty’s voice behavior, and vice versa.*

Building on Hypotheses 4 and 5, this study further posits that servant leadership may moderate the mediating effect of thriving at work. Specifically, in a high servant leadership organizational context, the trust, support, and inclusiveness demonstrated by leadership can increase university faculty’s instrumental and emotional resource pool, reducing the pressure from resource loss associated with voice behavior, thereby encouraging more voice behavior and strengthening the positive impact of thriving at work on voice behavior. Conversely, in a low servant leadership organizational context, voice behavior may pose significant accountability risks for university faculty, and to avoid responsibility and reduce resource loss, they tend to engage in less voice behavior, thereby weakening the positive impact of thriving at work on voice behavior and diminishing the mediating effect of thriving at work in the relationship between mission valence and voice behavior. Based on this, this study proposes Hypothesis 6:

**Hypothesis** **6.** *Servant leadership positively moderates the indirect effect of mission valence on university faculty’s voice behavior through thriving at work, with this indirect effect being stronger under conditions of high servant leadership than under low servant leadership.*

### 2.5. The Research Model of Study

**Figure 1:**
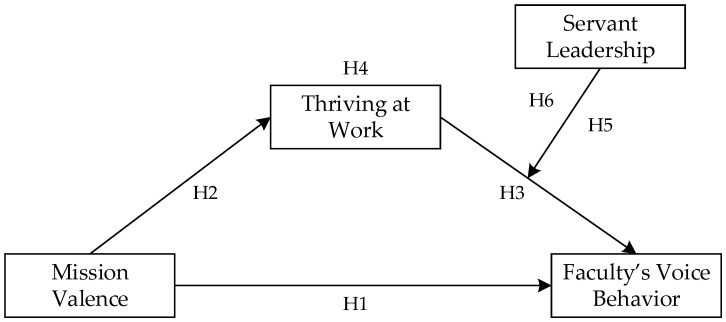
The research model of the study.

Based on the above theoretical derivation and analysis, this study aims to explore how mission valence influences university faculty’s voice behavior. The theoretical model of this study is shown in Figure 1.

## 3. Methods

### 3.1. Data Collection and Sample

In selecting the sample, considering the abundant and diverse resources in higher education institutions in Hubei Province, where the author is based, this study targeted university faculty in Hubei Province. Specifically, the study selected almost 70 higher education institutions as representatives, including Wuhan University, Huazhong University of Science and Technology, China University of Geosciences (Wuhan), Wuhan University of Technology, Huazhong Agricultural University, Central China Normal University, Zhongnan University of Economics and Law, Yangtze University, Hubei Minzu University, Jianghan University, and Three Gorges University. The research subjects included teaching staff within these universities. Questionnaires were distributed and collected both online and offline. To reduce common method bias, the study administered questionnaires in two waves, with a one-month interval. The first wave measured basic information, mission valence, and servant leadership, distributing 710 questionnaires and receiving 689 responses. After excluding those with insufficient response time and excessive missing data, 671 valid samples were obtained. The second wave measured thriving at work and voice behavior, with 673 responses received. After similar exclusions, 662 valid questionnaires were obtained, successfully matching 630 respondents across both waves, resulting in an effective response rate of 88.7%.

### 3.2. Demographic Information

The demographic information of the valid sample is presented in Table 1. The gender ratio among university faculty shows a disparity but remains relatively balanced overall, with a higher proportion of female faculty (375 individuals, accounting for 59.5% of the total) compared to male faculty (255 individuals, accounting for 40.5%). Most respondents were young and middle-aged faculty, with 82 individuals (13%) aged 25 and below, 360 individuals (57.1%) aged 26–35, 112 individuals (17.8%) aged 36–45, 61 individuals (9.7%) aged 46–55, and 15 individuals (2.4%) aged 56 and above. The educational level of university faculty was relatively high, with all respondents holding at least a bachelor’s degree. Faculty with less than 3 years of experience accounted for 34.8% (219 individuals), while those with more than 3 years of experience accounted for 65.2% (411 individuals), including 147 individuals with 4–6 years of experience, 86 individuals with 7–10 years of experience, and 178 individuals with more than 10 years of experience. Overall, the surveyed university faculty had stable positions and relatively rich work experience, making them suitable for exploring the mechanisms of influence between mission valence and voice behavior.

### 3.3. Variable Measurement

This study adopted a deductive reasoning research paradigm, using quantitative statistical analysis methods for hypothesis testing. Therefore, all variables were assessed using scales that have been validated in the literature and widely used in the past, with specific entries as described in Table 2. We employed standard translation and back-translation procedures and relevant suggestions to translate items from English into Chinese [71]. All scales used five-point Likert scales (1 = strongly disagree, 5 = strongly agree), with participants reporting their agreement with each item.

#### 3.3.1. Mission Valence

Mission valence was measured using three items developed by Wright (2007) [11], with one item being “This division provides valuable public services”.

#### 3.3.2. Thriving at Work

Thriving at work was assessed using a 10-item scale developed by Porath et al. (2012) [72], with a sample item being “I find myself learning often”.

#### 3.3.3. Servant Leadership

Servant leadership was measured using a six-item scale developed by Sendjaya et al. (2019) [73], with a sample item being “Uses power in service to others, not for his or her ambition”.

#### 3.3.4. Faculty’s Voice Behavior

Faculty’s voice behavior was assessed using a 10-item scale developed by Liang et al. (2012) [74], with a sample item being “Proactively develop and make suggestions for issues that may influence the unit”.

#### 3.3.5. Control Variables

Previous research [75,76] has included gender, age, education level, and tenure as control variables because of their potential impact on employee voice behavior. Consistent with this approach, we also controlled for demographic variables such as gender, age, education level, and tenure to minimize the risk of misleading associations.

## 4. Results

### 4.1. Reliability and Validity

Reliability and validity are important indicators for evaluating the quality of research measurement tools. Reliability refers to the stability and consistency of a measurement tool, meaning that the measurement results remain relatively stable and consistent when applied at different times, in different environments, or by different individuals. Common methods for reliability analysis include test-retest, split-half, and reliability coefficients. Validity refers to whether a measurement tool accurately measures the concept, variable, or phenomenon being studied. Common methods for validity analysis include external validity, internal validity, content validity, and response validity. This study employed a three-step process to analyze the reliability and validity of the questionnaire. First, Cronbach’s alpha was used to assess reliability. Generally, a Cronbach’s alpha greater than 0.7 is considered acceptable, greater than 0.8 is considered good, and greater than 0.9 is considered excellent. As shown in Table 2, the Cronbach’s alpha values for each construct ranged from 0.900 to 0.969, with an overall Cronbach’s alpha of 0.979, indicating that the reliability met the standard.

Second, the results of the Kaiser–Meyer–Olkin (KMO) measure and Bartlett’s test of sphericity, which evaluate the adequacy and correlation of the data, were analyzed. The KMO measure assessed the correlation between variables, with values ranging from 0 to 1, and values closer to 1 indicating stronger correlations between variables. In the analysis results, the KMO value was 0.971, suggesting that the sample data were well-suited to analysis, with high correlations between variables. Bartlett’s test of sphericity tests whether the correlations between variables are significant. The test provides an approximate chi-square value, which, along with degrees of freedom (*df*) and significance level (sig.), provides the test results. In the analysis results, Bartlett’s test of sphericity yielded an approximate chi-square value of 22,802.387, with degrees of freedom of 406, and a significance level of 0.000. Together, the sample data showed good fit and correlation in the KMO measure and Bartlett’s test of sphericity, indicating that the data were suitable for further analysis and research.

Third, we conducted a confirmatory factor analysis (CFA) using Amos 24.0 to test whether the data fitted our hypothesized measurement model and to evaluate the validity of the measurements. We compared the *χ*^2^, *χ*^2^/*df*, GFI, AGFI, RMR, RMSEA, and CFI values of the single-factor model, two-factor model, three-factor model, and four-factor model to assess the fit (see Table 3). The results indicated that the four-factor model comprising mission valence, thriving at work, servant leadership, and faculty’s voice behavior achieved satisfactory fit levels, with all indicators significantly outperforming other models, suggesting that the four core variables in this study exhibit good discriminant validity. Additionally, we analyzed the values of composite reliability (CR) and average variance extracted (AVE) (ranging from 0.939 to 0.974 and 0.728 to 0.837, respectively), which exceeded the required thresholds (0.70 and 0.50, respectively), supporting the convergent validity of the constructs.

### 4.2. Descriptive Statistics and Correlation Analysis

Table 4 shows the means, standard deviations, and correlations among the study variables. The results indicate a significant positive correlation between mission valence and university faculty’s voice behavior (r = 0.613, *p* < 0.001), suggesting that mission valence positively influences university faculty’s voice behavior. A positive correlation was also found between mission valence and thriving at work (r = 0.698, *p* < 0.001), indicating that mission valence may enhance the psychological state of university faculty and thus promote voice behavior. The correlation between thriving at work and university faculty’s voice behavior was also positive (r = 0.780, *p* < 0.001), indicating a significant positive relationship between thriving at work and university faculty’s voice behavior. Additionally, a significant positive correlation was observed between servant leadership and voice behavior (r = 0.793, *p* < 0.001). Together, these correlation analyses demonstrate the complex interrelationships among mission valence, thriving at work, servant leadership, and university faculty’s voice behavior, providing preliminary support for the study’s hypotheses.

### 4.3. Hypothesis Testing

#### 4.3.1. Direct and Mediating Effects

This study utilized SPSS 27.0 statistical software to conduct hierarchical regression analysis on the relationship between mission valence and faculty’s voice behavior in higher education institutions. The results, as shown in Table 5, indicate that mission valence has a significant positive impact on faculty’s voice behavior (*β* = 0.549, *p* < 0.001), explaining 30.6% of the variance in voice behavior after controlling for other variables, thus supporting Hypothesis 1. Model 2 shows that mission valence significantly positively influences thriving at work (*β* = 0.711, *p* < 0.001), accounting for 42.6% of the variance in thriving at work after controlling for other variables, supporting Hypothesis 2. Model 5 demonstrates that thriving at work significantly positively impacts faculty’s voice behavior (*β* = 0.604, *p* < 0.001), explaining 51.8% of the variance in voice behavior after controlling for other variables, supporting Hypothesis 3.

This study employed the mediating test method proposed by Baron and Kenny to examine whether thriving at work mediates the relationship between mission valence and voice behavior [77]. Models 2, 5, and 6 show that when both mission valence and thriving at work predict faculty’s voice behavior, the regression coefficients for both mission valence (*β* = 0.119, *p* < 0.001) and thriving at work (*β* = 0.604, *p* < 0.001) are significant. However, the regression coefficient for mission valence on faculty’s voice behavior decreased from 0.549 to 0.119, indicating that thriving at work partially mediates the relationship between mission valence and faculty’s voice behavior, thus supporting Hypothesis 4. To further rigorously test the mediating effect, this study used the SPSS 27.0 Process program to conduct bootstrap testing with 5000 repeated samples. The results showed that the mediating effect of thriving at work was 0.430, with a 95% confidence interval ([0.373, 0.495]) that did not include 0, indicating a significant mediating effect of thriving at work, further supporting Hypothesis 4.

#### 4.3.2. Moderating Effects

**Figure 2:**
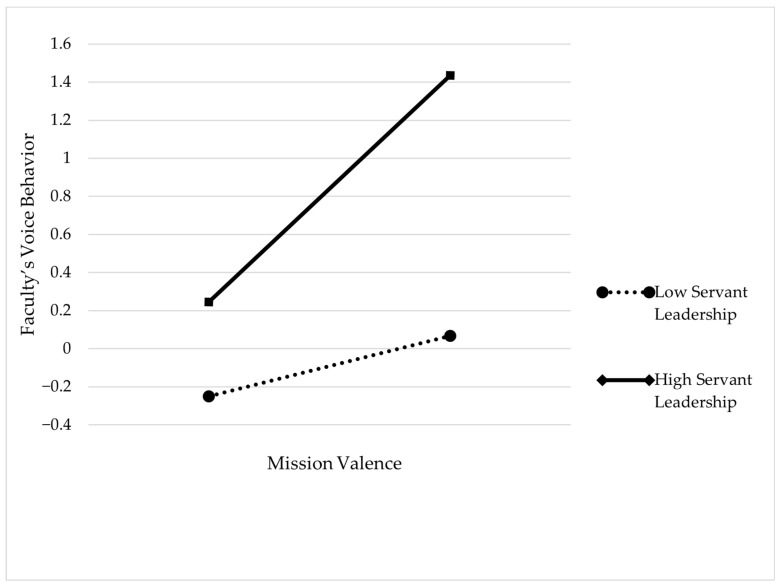
Moderating effect of servant leadership.

This study utilized hierarchical regression analysis in SPSS 27.0 to test the moderating effect of servant leadership on the relationship between thriving at work and faculty’s voice behavior. In the first step, the control variables were regressed onto voice behavior. In the second step, thriving at work was regressed onto voice behavior. In the third step, the moderating variable of servant leadership was added. In the fourth step, the interaction term between thriving at work and servant leadership, after centralization, was added. As shown in Table 6, the interaction term between thriving at work and servant leadership significantly predicts voice behavior (*β* = 0.218, *p* < 0.001), indicating that servant leadership positively moderates the relationship between thriving at work and voice behavior, thus supporting Hypothesis 5. Figure 2 illustrates the moderating effect of servant leadership on the relationship between thriving at work and faculty’s voice behavior, showing that the stronger the servant leadership, the stronger the positive impact of thriving at work on faculty’s voice behavior.

This study used the bootstrap method in SPSS 27.0 Process program to test the moderated mediation effect, with 5000 repeated samples. The results in Table 7 show that when servant leadership was low, the indirect effect of mission valence on faculty’s voice behavior through thriving at work was 0.139, with a confidence interval ([0.065, 0.211]); when servant leadership was high, the indirect effect was 0.394, with a confidence interval ([0.323, 0.468]). Since the confidence intervals did not include 0, it is evident that the indirect effect of mission valence on faculty’s voice behavior through thriving at work was significant regardless of the level of servant leadership, while servant leadership intensified the mediating role of thriving at work. Additionally, the INDEX values and other indicators in Table 7 reveal that servant leadership moderated the indirect effect of mission valence on faculty’s voice behavior through thriving at work, with an INDEX value of 0.162 and a confidence interval ([0.127, 0.201]) that did not include 0, indicating that the moderated mediation effect of servant leadership was confirmed, thus supporting Hypothesis 6.

## 5. Discussion and Implications

### 5.1. Discussion

Based on conservation of resources theory and the broaden-and-build theory of positive emotions, this study proposed a moderated mediation model to explore the associations and boundary conditions between mission valence and faculty’s voice behavior in higher education institutions. By analyzing empirical data, we examined how and under what conditions mission valence is linked to faculty’s voice behavior in higher education. The main findings are as follows:

First, the results indicate a significant positive association between mission valence and faculty’s voice behavior in higher education, as hypothesized. Previous research primarily explored the impact of mission valence on work attitudes and motivation [12,78], but empirical studies examining its link to proactive behavior have yet to be fully developed. Similarly, existing research has mainly focused on organizational-level factors such as leadership types influencing voice behavior [75], with less attention given to individual-level predictors of voice behavior. Our findings highlight a strong correlation between mission valence and faculty’s voice behavior in higher education. Thus, this study expands the understanding of the associations between mission valence and voice behavior.

Second, to better understand how mission valence is linked to faculty’s voice behavior, we further explored the internal linkage mechanisms between mission valence and voice behavior. Drawing on conservation of resources theory and the broaden-and-build theory of positive emotions, the findings suggest that thriving at work partially mediates the relationship between mission valence and faculty’s voice behavior, highlighting that thriving at work is an important factor connecting mission valence and voice behavior. This study reveals that mission valence is positively associated with faculty’s thriving at work, which, in turn, positively correlates with faculty’s voice behavior. Therefore, this study underscores the important role of individual cognition in fostering thriving at work, consistent with previous research [79].

Third, this study examined the boundary conditions of the association between mission valence and faculty’s voice behavior. The results show that servant leadership positively moderates the relationship between thriving at work and faculty’s voice behavior and moderates the indirect association between mission valence and voice behavior through thriving at work. Previous research has found that although individuals can influence their personal outcomes through thriving at work, it is also shaped by the organizational environment [80]. Existing literature has examined various contextual variables moderating the relationship between thriving at work and personal outcomes, such as family-supportive supervisor behavior [81] and trust climate [82]. This study, focusing on university faculty, considers the importance of servant leadership in higher education reforms and explores the moderating role of servant leadership in the relationship between thriving at work and faculty’s voice behavior, offering new insights into understanding thriving at work and expanding the discussion on the supportive contexts associated with thriving at work on university faculty.

### 5.2. Theoretical Contributions

The findings of this study contribute to the development of theories related to mission valence and voice behavior in several ways.

First, this study contributes to the literature on organizational voice behavior. Recently, scholars have called for more attention to the predictors of organizational voice behavior. In response to this call, we focused on examining the relationship between mission valence and faculty’s voice behavior in higher education. Previous research has shown that different individual cognitive factors are associated with faculty’s voice behavior [7,9]. However, as an important cognitive factor [22], the role of mission valence has not been fully explored. Our study provides empirical evidence that university faculty’s perception of the importance of the organizational mission, i.e., mission valence, is significantly associated with their voice behavior. This finding offers an important supplement to the literature on organizational voice behavior and expands the theoretical research perspective. Additionally, our empirical research responds to the call by other scholars to explore how mission valence is linked to employees’ extra-role and proactive behaviors [15].

Second, this study uncovers the internal transmission mechanism through which mission valence is related to faculty’s voice behavior. Combining conservation of resources theory and the broaden-and-build theory of positive emotions, this study suggests that thriving at work can effectively mediate the association between mission valence and faculty’s voice behavior, providing a new perspective for understanding the psychological mechanisms linking mission valence to faculty’s voice behavior in higher education. This finding responds to researchers’ calls for deeper analysis of the psychological mechanisms behind faculty’s voice behavior [5] and highlights the potential role of thriving at work in the relationship between individual cognition and behavioral performance [26,83].

Third, this study helps reveal the moderating role of servant leadership on the relationship between thriving at work and faculty’s voice behavior and its moderating effect on the entire mediation mechanism of mission valence–thriving at work voice behavior. By using servant leadership as a moderating variable, this study shows that whether university faculty engage in voice behavior is not only influenced by their thriving at work but also by their department leadership. The leadership style plays an even more critical role. Only when university faculty perceive high servant leadership does the association between thriving at work and voice behavior become significantly stronger, and the relationship between mission valence and voice behavior through thriving at work is more pronounced. This finding aligns with existing conclusions about the relationship between servant leadership and employee voice behavior in both the public and private sectors [84,85,86]. Moreover, it responds to researchers’ calls for greater attention to the boundary conditions of proactive behavior [87]. Additionally, it provides empirical evidence supporting existing theoretical assumptions about promoting voice behavior [88].

### 5.3. Managerial Implications

In addition to the theoretical contributions mentioned above, this study may have several managerial implications.

First, continuously enhancing faculty’s mission valence through the education activity series upholding the spirit of “remain true to our original aspiration and keep our mission firmly in mind” within higher education institutions is crucial. Mission valence is a vital factor in motivating faculty in the public sector to take responsibility and actively engage in voice behavior. The findings of this study indicate that mission valence can, to a large extent, positively predict faculty’s thriving at work and voice behavior in higher education. Therefore, higher education institutions should focus on enhancing faculty’s mission valence by continuously conducting mission-themed educational activities through daily training, expert lectures, and establishing role model figures, promoting faculty’s understanding and recognition of the mission and goals during educational reform, enhancing their sense of mission responsibility, and thus fostering their positive work state and voice behavior.

Second, attention should be given to cultivating and enhancing faculty’s thriving at work in higher education. Thriving at work is one of the key factors in promoting faculty’s active voice behavior. Thriving at work not only directly shows a positive association with faculty’s voice behavior but also serves as an important mediator in the relationship between mission valence and faculty’s voice behavior in higher education. Therefore, during faculty recruitment, assessment, and promotion processes, attention should be paid to evaluating their thriving at work. Additionally, the findings of this study suggest that thriving at work is dynamic. Higher education institutions can enhance faculty’s work vitality and proactive learning consciousness through continuous mission education, provide work resources support, and strengthen their willingness and ability to continually improve themselves, thus enhancing their thriving at work and further enhancing the association between mission valence and voice behavior.

Third, strengthening the development of servant leadership in higher education institutions is essential. High levels of servant leadership are significant factors influencing faculty’s willingness to engage in voice behavior. This study found that when servant leadership levels are high in higher education, the association between thriving at work and faculty’s voice behavior becomes stronger, and the relationship between mission valence and voice behavior through thriving at work is more pronounced, while low servant leadership levels counteract the motivating effect of thriving at work on voice behavior and diminish the connection between mission valence and voice behavior through thriving at work. Therefore, to stimulate faculty’s voice behavior, higher education departments should not only enhance faculty’s thriving at work at the individual level but also strengthen the development of servant leadership at the organizational level. Creating a relaxed environment for faculty’s voice behavior, making it a low-risk responsibility and obligation beneficial to the development of higher education institutions, guiding and encouraging them to continuously communicate and learn, and thereby enhancing their ability to engage in voice behavior, ensures that those who wish to voice their opinions have the courage to do so.

### 5.4. Limitations and Future Research

This study has several limitations. First, the use of cross-sectional data to test the hypotheses does not reflect the causal relationship between mission valence and faculty’s voice behavior in higher education institutions. Additionally, although the sample size met the requirements for statistical analysis, the findings may still lack some persuasion. Future research could adopt longitudinal or follow-up data collection methods with larger samples to examine the causal relationship between mission valence and voice behavior. In addition, there is a large number of administrators in Chinese universities who are responsible for the daily management and technical support of their schools, among other things. The difference between them and the teaching faculty group is also an important future research direction.

Second, the study used self-report data, making it difficult to completely avoid common method bias and social desirability bias. Future research could combine self-reports with other-report methods or employ experimental methods to reduce common method bias and social desirability bias, thereby further enhancing the reliability of the findings.

Third, this study analyzed the moderating effect of servant leadership based on individual perceptions, which may not accurately reflect the organizational factors influencing faculty’s voice behavior in higher education institutions. Future research could employ cross-level analysis methods, considering both individual perception and organizational levels, to construct an integrated model of factors influencing faculty’s voice behavior in higher education institutions.

## 6. Conclusions

This study combined conservation of resources theory and the broaden-and-build theory of positive emotions to propose a moderated mediation model. It examined whether and how mission valence influences faculty’s voice behavior in higher education institutions, which is valuable in the context of education activity series upholding the spirit of “remain true to our original aspiration and keep our mission firmly in mind”. The empirical results indicate that mission valence has a significant positive impact on faculty’s voice behavior. Thriving at work plays a mediating role in this relationship. Additionally, servant leadership plays a positive moderating role. These findings provide insights into how to enhance mission valence among faculty in higher education institutions and thereby encourage more voice behavior.

## Figures and Tables

**Table 1:** Sample demographics (*n* = 630).

Characteristic	Classification	Frequency	Percentage
Gender	Male	255	40.5%
	Female	375	59.5%
Age	25 years old or below	82	13.0%
	26–35	360	57.1%
	36–45	112	17.8%
	46–55	61	9.7%
	56 years old or above	15	2.4%
Education	Associate degree or below	86	13.7%
	Bachelor’s degree	440	69.8%
	Master’s degree or above	104	16.5%
Tenure	1 year or below	56	8.9%
	1–3 years	163	25.9%
	4–6 years	147	23.3%
	7–10 years	86	13.7%
	10 years or above	178	28.2%

**Table 2:** Reliability and Validity Analysis (*n* = 630).

Construct and Its Items	CITC	Cronbach’s α
Mission Valence (AVE = 0.837; CR = 0.939)		
1. This division provides valuable public services.	0.774	0.900
2. The work of this division is very significant in the broader scheme of things.	0.842	
3. I believe that the priorities of this division are quite important.	0.799	
Thriving at Work (AVE = 0.788; CR = 0.974)		
1. I find myself learning often.	0.785	0.969
2. I continue to learn more and more as time goes by.	0.845	
3. I see myself continually improving.	0.863	
4. I have developed a lot as a person.	0.896	
5. I am learning.	0.840	
6. I feel alive and vital.	0.903	
7. I have energy and spirit.	0.893	
8. I feel very energetic.	0.838	
9. I feel alert and awake.	0.858	
10. I am looking forward to each new day.	0.871	
Servant Leadership (AVE = 0.795; CR = 0.959)		
1. Uses power in service to others, not for his or her ambition.	0.793	0.947
2. Gives me the right to question his or her actions and decisions.	0.887	
3. Respects me for who I am, not how I make him or her feel.	0.839	
4. Enhances my capacity for moral actions.	0.832	
5. Helps me to generate a sense of meaning out of everyday life at work.	0.845	
6. Contributes to my personal and professional growth.	0.863	
Faculty’s Voice Behavior (AVE = 0.728; CR = 0.964)		
1. Proactively develop and make suggestions for issues that may influence the unit.	0.769	0.956
2. Proactively suggest new projects which are beneficial to the work unit.	0.818	
3. Raise suggestions to improve the unit’s working procedure.	0.850	
4. Proactively voice out constructive suggestions that help the unit reach its goals.	0.850	
5. Make constructive suggestions to improve the unit’s operation.	0.859	
6. Advise other colleagues against undesirable behaviors that would hamper job performance.	0.857	
7. Speak up honestly with problems that might cause serious loss to the work unit, even when/though dissenting opinions exist.	0.805	
8. Dare to voice out opinions on things that might affect efficiency in the work unit, even if that would embarrass others.	0.779	
9. Dare to point out problems when they appear in the unit, even if that would hamper relationships with other colleagues.	0.761	
10. Proactively report coordination problems in the workplace to the management.	0.787	

**Table 3:** Confirmatory factor analysis (*n* = 630).

Model	Factor	$\chi^{2}$	df	$\chi^{2}/df$	RMSEA	SRMR	CFI	IFI
Four-factor model	MV; TW; SL; FVB	1247.858	341	3.659	0.065	0.0384	0.960	0.960
Three-factor model	MV; TW; SL + FVB	4750.122	374	12.701	0.136	0.0591	0.808	0.808
Two-factor model	MV; TW + SL + FVB	6329.523	376	16.834	0.159	0.0694	0.739	0.739
One-factor model	MV + TW + SL + FVB	6886.768	377	18.267	0.166	0.0731	0.714	0.715

Note. MV = mission valence. FVB = faculty’s voice behavior. TW = thriving at work. SL = servant leadership.

**Table 4:** Means, standard deviations, and correlations among study variables (*n* = 630).

Variables	1	2	3	4	5	6	7	8
1. Gender	1							
2. Age	−0.155 ***	1						
3. Education	0.025	−0.285 ***	1					
4. Tenure	−0.020	0.732 ***	−0.266 ***	1				
5. MV	−0.112 **	0.197 ***	−0.098 *	0.187 ***	1			
6. FVB	−0.161 ***	0.272 ***	−0.152 ***	0.261 ***	0.613 ***	1		
7. TW	−0.126 **	0.236 ***	−0.155 ***	0.211 ***	0.698 ***	0.780 ***	1	
8. SL	−0.121 **	0.197 ***	−0.137 ***	0.184 ***	0.691 ***	0.793 ***	0.809 ***	1
*Mean*	1.600	2.310	2.030	3.270	4.467	4.212	4.252	4.232
*SD*	0.491	0.902	0.549	1.347	0.755	0.728	0.800	0.810

Note. MV = mission valence. FVB = faculty’s voice behavior. TW = thriving at work. SL = servant leadership. * *p* < 0.05, ** *p* < 0.01, *** *p* < 0.001.

**Table 5:** Analysis of the mediating effect of thriving at work (*n* = 630).

Variables	Thriving at Work		Faculty’s Voice Behavior			
	Model 1	Model 2	Model 3	Model 4	Model 5	Model 6
Gender	−0.166 *	−0.062	−0.205 ***	−0.125 **	−0.092 *	−0.087 *
Age	0.112 *	0.057	0.096 *	0.053	0.020	0.019
Education	−0.134 *	−0.095 *	−0.098	−0.068	−0.007	−0.010
Tenure	0.055	0.012	0.082 **	0.049 *	0.045 *	0.042 *
Mission Valence		0.711 ***		0.549 ***	0.682 ***	0.119 ***
Thriving at Work						0.604 ***
$R^{2}$	0.076	0.503	0.105	0.412	0.623	0.631
${\Delta R}^{2}$	0.076 ***	0.426 ***	0.105 ***	0.306 ***	0.518 ***	0.219 ***
F	12.906 ***	126.164 ***	18.422 ***	87.424 ***	206.498 ***	177.610 ***

Note. * *p* < 0.05, ** *p* < 0.01, *** *p* < 0.001.

**Table 6:** Hierarchical regression results (*n* = 630).

Variables	Faculty’s Voice Behavior			
	M1	M2	M3	M4
Gender	−0.205 ***	−0.092 *	−0.077 *	−0.034
Age	0.096 *	0.020	0.025	0.014
Education	−0.098	−0.007	0.000	0.031
Tenure	0.082 **	0.045 *	0.040 *	0.032
Thriving at Work		0.682 ***	0.344 ***	0.377 ***
Servant Leadership			0.416 ***	0.466 ***
Thriving at Work × Servant Leadership				0.218 ***
$R^{2}$	0.105	0.623	0.697	0.738
${\Delta R}^{2}$	0.105 ***	0.518 ***	0.074 ***	0.040 ***
F	18.422 ***	206.498 ***	239.055 ***	249.724 ***

Note. * *p* < 0.05, ** *p* < 0.01, *** *p* < 0.001.

**Table 7:** Moderated mediation effect testing results (*n* = 630).

Servant Leadership	Conditional Indirect Effects				Moderated Mediation Effects			
	Effect	SE	LLCI	ULCI	INDEX	SE	LLCI	ULCI
Low	0.139	0.037	0.065	0.211	0.162	0.019	0.127	0.201
High	0.394	0.037	0.323	0.468				

## Data Availability

The data presented in this study are available on request from the corresponding author. The data are not publicly available due to confidentiality and research ethics.
